# The influence of marathon running on resting-state EEG activity: a longitudinal observational study

**DOI:** 10.1007/s00421-023-05356-4

**Published:** 2023-11-29

**Authors:** Joanna Moussiopoulou, Benjamin Pross, Mirjam Handrack, Daniel Keeser, Oliver Pogarell, Martin Halle, Peter Falkai, Johannes Scherr, Alkomiet Hasan, Astrid Roeh

**Affiliations:** 1 Department of Psychiatry and Psychotherapy, LMU University Hospital, LMU Munich, Nußbaumstraße 7, 80336 Munich, Germany; 2 Department of Prevention and Sports Medicine, University Hospital Klinikum Rechts Der Isar, Technical University Munich, Georg-Brauchle-Ring 56, 80992 Munich, Germany; 3 DZHK (German Center for Cardiovascular Research, Partner Site Munich Heart Alliance), Potsdamer Str. 58, 10785 Berlin, Germany; 4 University Center for Preventive and Sports Medicine, Balgrist University Hospital, University of Zurich, Forchstrasse 340, 8008 Zurich, Switzerland; 5 Department of Psychiatry, Psychotherapy and Psychosomatics of the University Augsburg, Medical Faculty, Bezirkskrankenhaus Augsburg, University of Augsburg, Augsburg, Germany; 6 NeuroImaging Core Unit Munich (NICUM), Ludwig Maximilian University Munich, Nußbaumstraße 7, 80336 Munich, Germany

**Keywords:** Exercise, Plasticity, Neuroplasticity, Running, Physical activity, Electrocortical activity, Electroencephalography

## Abstract

Physical activity (PA) has positive effects on various health aspects and neuronal functions, including neuronal plasticity. Exceeding a certain exercise frequency and duration has been associated with negative effects. Our study investigated the effects of excessive PA with a marathon run (MA) and regular PA (training and recovery phases) on electrocortical activity, as measured by electroencephalography (EEG). Thirty healthy marathon runners (26 male, 45 ± 9 yrs) were enrolled in the study. Four resting-state 32 channel EEG recordings were conducted: 12–8 weeks before MA (T-1), 14–4 days prior to MA (T0), 1–6 days after (T2), and 13–15 weeks after MA (T3). Power spectrum analyses were conducted using standardized Low-Resolution Electromagnetic Tomography (sLORETA) and included the following frequency bands: delta (1.5–6 Hz), theta (6.5–8.0 Hz), alpha1 (8.5–10 Hz), alpha2 (10.5–12.0 Hz), beta1 (12.5–18.0 Hz), beta2 (18.5–21.0 Hz), beta3 (21.5–30.0 Hz), and total power (1.5-30 Hz). Statistical nonparametric mapping showed reduced power both in the alpha-2 (log-F ratio = − 0.705, threshold log-F ratio =  ± 0.685, *p* < 0.05) and in the delta frequency band (log-F ratio = −0.699, threshold log-F ratio =  ± 0.685, *p* < 0.05) in frontal cortical areas after MA (T2 vs. T0). These effects diminished at long-term follow-up (T3). The results can be interpreted as correlates for subacute neuroplasticity induced by strenuous and prolonged PA. Although previous studies reported an increase in alpha frequency during and directly postexercise, the adverse observation a few days after exercise cessation suggests counterregulatory mechanisms, whose complex origin can be suspected in subcortical circuits, changes in neurotransmitter systems and modulation of affectivity.

## Introduction

Physical activity (PA) and aerobic exercise in particular have been repeatedly associated with various health benefits. Especially well documented are positive effects on cardiovascular aspects, such as hypertension (Diaz and Shimbo 2013; Dimeo et al. 2012), coronary heart disease (Crawford and Morris 1958; Sesso et al. 2000), the metabolic system (Ivy 1997; Kirwan et al. 2017), tumor (Delbruck 2012; Frisch et al. 1985) and overall mortality (Schnohr et al. 2015).

There is increasing evidence of the beneficial effects of PA on neurological and psychiatric diseases, such as dementia, depression and schizophrenia (Emrah Duzel et al. 2016; Falkai et al. 2021; Oertel-Knochel et al. 2014; Schuch et al. 2018). As a result, PA has been included in therapeutic regimens in these cases (Malchow et al. 2013), although the mechanisms behind these observations are not yet fully understood. The induction and modulation of neuroplasticity seems to play a key role in these effects. PA increases cortical capillary supply, induces the development of new neurons (Colcombe et al. 2004), causes alterations in cytoarchitecture, increases cellular proliferation, dendritic complexity (Eadie et al. 2005) and the release of neurotrophic factors such as brain-derived neurotrophic factor (BDNF) (Emrah Duzel et al. 2016). BDNF, in turn, has been positively correlated with an increase in hippocampal volume (Erickson et al. 2012; McKee et al. 2014). In this regard, PA leads to an increase and maintenance of hippocampal volume and thus may support the prevention of cognitive decline and memory loss. This modulation of neuroplasticity also leads to increased cortical connectivity and activation, which has been confirmed in functional MRI studies (Colcombe et al. 2004; Voss et al. 2010).

Regarding the representation of these PA-induced neuroplasticity effects in electroencephalography (EEG), consistent findings are lacking. The previous studies have mainly focused on changes in EEG frequencies during PA and a few minutes after cessation of PA, allowing conclusions to be drawn about acute effects but less about subacute or chronic effects. Moreover, the previous studies mainly focused on certain frequency bands, specific cortical areas, and moderate activity or a single short bout of exercise, termed “acute exercise” (Basso and Suzuki 2017). To our knowledge, there are no studies on neurophysiological effects of such strenuous and prolonged PA as a marathon, demonstrated with EEG. Previous findings showed that strenuous exercise leading to dehydration impairs both information processing and memory functions (Tomporowski 2003). Consistent with negative impact on other health domains (e.g., cardiac repolarization, electrolyte status (Scherr et al. 2012)), strenuous exercise might also have similar detrimental effects on neuroplasticity or electrocortical activity.

Because the plasticity is assumed to represent a subacute and chronic process, a certain time interval between PA cessation and recordings is of great importance. Other neuropsychological outcomes, such as cognitive performance, were shown to improve with a latency of 24 h after marathon running as demonstrated in another study of the ReCaP trial (Roeh et al. 2021).

A common acute effect of PA in the EEG appears to be increased activity, or frontal hemispheric asymmetry, in the alpha frequency band (8–12 Hz), which could be interpreted as an indication of relaxation or a change in affect (Crabbe and Dishman 2004). Possible underlying mechanisms included enhanced somatosensory afferents (Krause et al. 1983; Youngstedt et al. 1993 FEB 01:), changes in norepinephrine concentrations (Stock et al. 1996), hypothalamic regulation (Nielsen et al. 2001), and modulation by subcortical regions such as the thalamus (Larson et al. 1998; Lindgren et al. 1999). However, most of these studies were old, and investigated the cortical effects of moderate PA.

This is the first study investigating the electrocortical effects of regular PA (during the preparation for a MA) and intense PA (MA) at four time points before (T-1, T0), after (T2) and during recovery (T3) from MA, with EEG in the source space carried out by sLORETA analyses. Moreover, the present study is not limited to specific frequency bands or cortical areas but provides current source density analyses covering all frequencies in the range 1.5–30 Hz and all cortical areas. Previous studies showed a temporary increase in alpha-power during and directly after PA cessation and a rapid decay of this effect. Owing to the high intensity of PA in our study, we expect to be able to detect the effect even several days after PA cessation (T2). This design allows us to present for the first time the EEG correlates of regular and intensive PA in a longitudinal design.

## Methods

### Participants and assessments

The participants were extracted from the study population of the ReCaP trial (Running effects on cognition and plasticity), a longitudinal cooperation study between the Clinic for Psychiatry and Psychotherapy at the LMU University of Munich and the Center for Prevention and Sports Medicine at the Technical University of Munich (Roeh et al. 2020).

Thirty healthy marathon runners participated in this longitudinal study. Inclusion criteria were experience in the field of endurance training (at least one completed half-marathon) as well as sufficient German language skills. Exclusion criteria included age < 18 or > 60 years, relevant pre-existing medical conditions, body mass index (BMI) ≥ 30 kg/m^2^ and consumption of cannabis. Written informed consent was obtained from each subject. All subjects underwent EEG recordings, demographic analysis (age, sex, smoking, handedness, total education, BMI) and the International Physical Activity Questionnaire (IPAQ). The latter is a validated (self-administered) measuring tool for the assessment of PA (Hagströmer et al. 2006), consisting of 27 questions regarding the previous 7 days’ activities according to domains (occupational PA, transportation PA, housework, house maintenance and caring for family, recreation, sport and leisure-time PA; and time spent sitting) (“Scoring protocol for the International Physical Activity Questionnaire (IPAQ)” 2023), and was assessed at the first visit.

### Declarations and ethics statement

The study was conducted in compliance with Good Clinical Practice guidelines, the guiding principles of the Declaration of Helsinki 2008 and local laws and regulations. Our study was reviewed and approved by the ethics committees of the Ludwig Maximilian University Munich Ethics committee (approval reference number 17-148) and the Technical University of Munich (approval reference number 218/17 S).

### EEG recordings

**Fig. 1:**
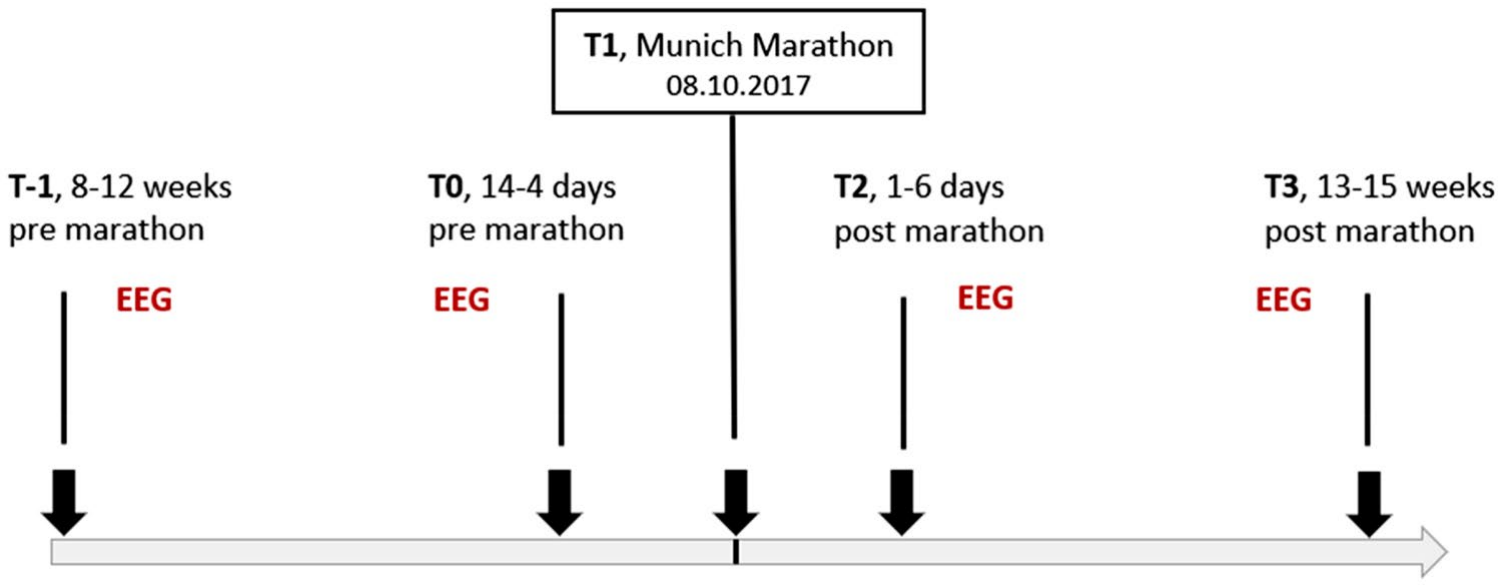
Time schedule of the EEG recordings

Resting-state EEG was recorded at four different visits around the Munich Marathon 2017, including the training and recovery periods (Fig. 1). The time between the first (T-1) and second (T0) recording represents the intensive training period. T0 represents the tapering phase of MA training where no intensive training took place. No EEG recordings took place on the Marathon day (08.10.2017, T1), as we could not guarantee the same setting of recordings at the marathon site compared to our laboratory, which would result in reduced comparability and we wanted to focus on the subacute and chronic adaptations. The first post-MA EEG recordings (T2) took place 1–6 days and the last recordings (T3) during the resting phase, 13–15 weeks after the marathon.

Resting-state EEG was assessed using electrode caps (“actiCap”, BrainProducts GmbH, Gilching) with 32 electrodes connecting the cap to the control box (“actiCap EEG active electrode system”), which was then connected to an amplifier (“BrainAmp S/N AMP0901983 Standard”). The electrodes were placed on the subject´s head in accordance with the international 10–20 system (Jasper 1958) at FP1, FP2, F7, F3, Fz, F4, F8, FC5, FC1, FC2, FC6, T7, C3, Cz, C4, T8, TP9, CP5, CP1, CP2, CP6, TP10, P7, P3, Pz, P4, P8, PO9, O1, Oz, O2 and PO10. Electrode skin impedance was less than 25 kΩ, which was achieved by application of a conductivity improving gel (“SuperVisc high-Viscosity Electrolyte-gel For Active Electrodes”, EASYCAP GmbH, Herrsching). Subjects were instructed to be in a resting yet wakeful state while sitting in a comfortably sitting position in a sound and light attenuated room in our laboratory at the university hospital LMU, department of psychiatry with eyes closed, for six-eight minutes. Subjects were supervised during the recording.

### Data processing

The EEG data were exported to BrainVision Analyzer Version 2.0 (Brain Products GmbH, Gilching) for further processing. A 70 Hz low-pass filter, a 1 Hz high-pass filter and a 50 Hz notch filter were used, along with a sampling rate of 250 Hz. Prior to data analysis, the artifacts were manually detected and removed (using the Raw Data Inspector function) to exclude eye movements and muscle contractions. From each recording 140 artifact-free EEG segments (one segment = 2000 ms) were used for further analysis and exported to the text ASCII format.

### Statistical analyses

#### Statistical analysis of demographic data

The demographic data (Table 1) were analyzed using SPSS (IBM SPSS Statistics, Version 26). Mean values and standard deviation (SD) were determined using descriptive statistics.

**Table 1:** Descriptive and anthropometric data for all subjects

Demographic findings	*N*		
Sex (m:f)	23:3		
Smoking (yes:no)	0:26		
Handedness (right:left)	24:2		

	*N*	Mean	SD
Age (years)	26	44.12	10.11
Education (years)	24	15.75	4.11
BMI (kg/m^2^)	26	23.22	2.35
IPAQ (MET min/week)	26	6485.94	5239.86
MA Running time (min)	22	226.95	26.35

*N* sample size, *m* male, *f* female, *BMI* Body mass index, *IPAQ* International Physical Activity Questionnaire, *MET* metabolic equivalent of task, *SD* Standard deviation

#### sLORETA analysis

**Fig. 2:**
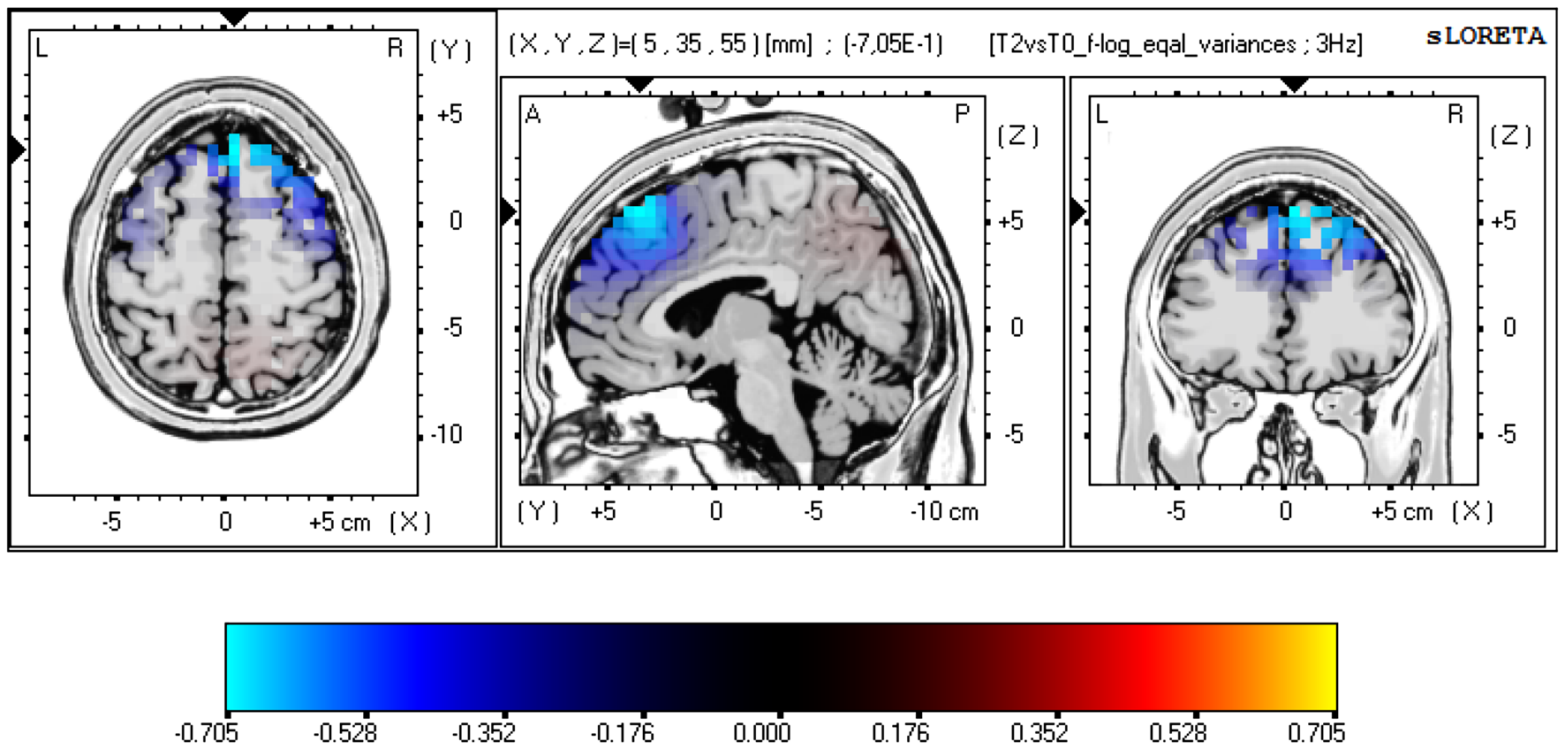
sLORETA slice viewer, T2 vs. T0: LORETA statistical maps of the alpha-2-frequency band; statistical non-parametric mapping (SnPM) of within-subject comparisons were performed to compare the current density distributions between T2 (T2 = 1–6 days after the marathon) and T0 (T0 = 14 to 4 days prior to the marathon); a significant reduction was detected for the alpha-2 power in T2 when compared to T0, maximum current density differences were localized in the right frontal cortex (*xyz* = 5, 35, 55; BA 8); the color bar represents the log-F-ratio value for each voxel

**Fig. 3:**
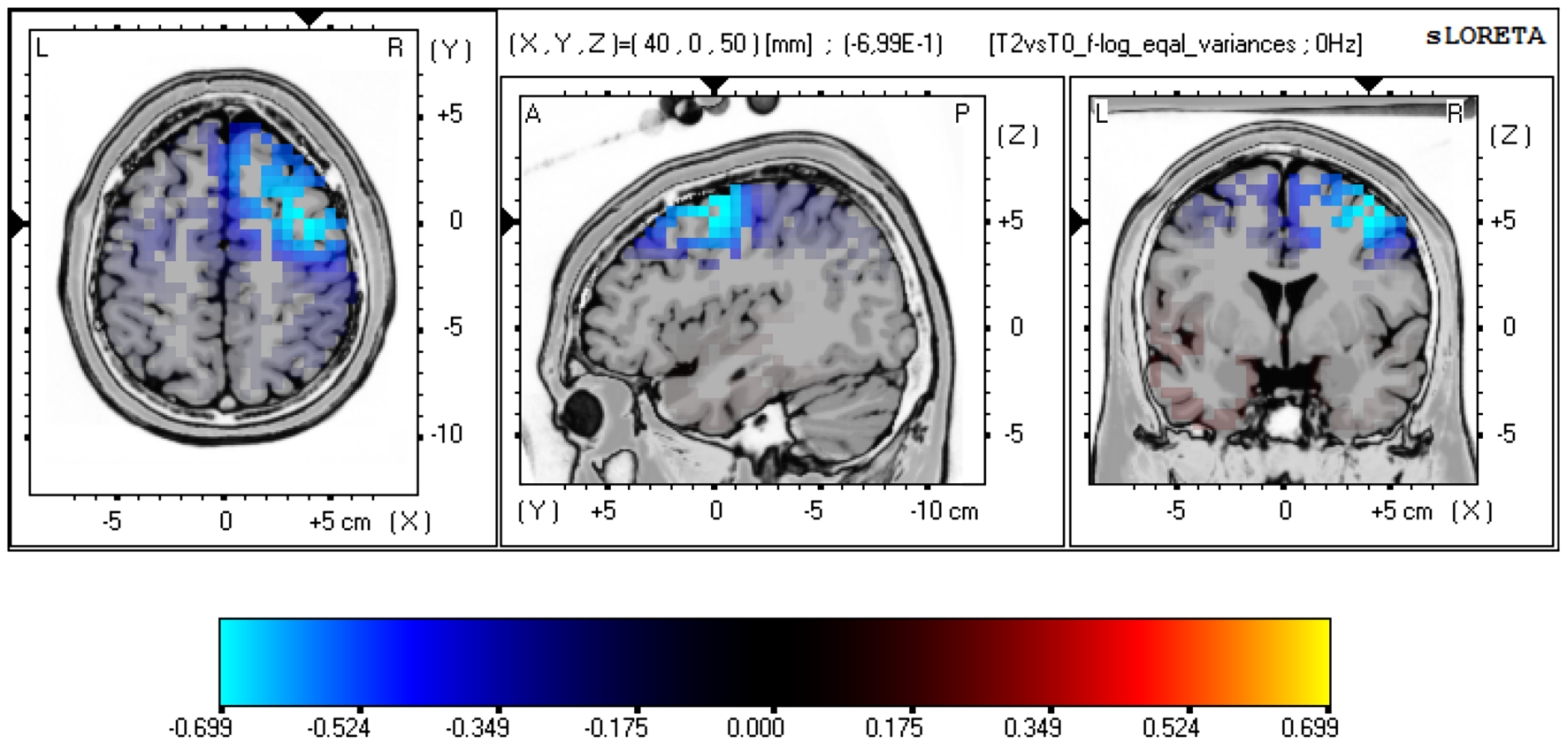
sLORETA slice viewer, T2 vs. T0: LORETA statistical maps of the delta-frequency band; statistical non-parametric mapping (SnPM) of within-subject comparisons were performed to compare the current density distributions between T2 (T2 = 1–6 days after the marathon) and T0 (T0 = 14 to 4 days prior to the marathon); a significant reduction was detected for the delta power in T2 when compared to T0, maximum current density differences were localized in the frontal cortex (*xyz* = 40, 0, 50; BA 6); the color bar represents the log-F-ratio value for each voxel

**Fig. 4:**
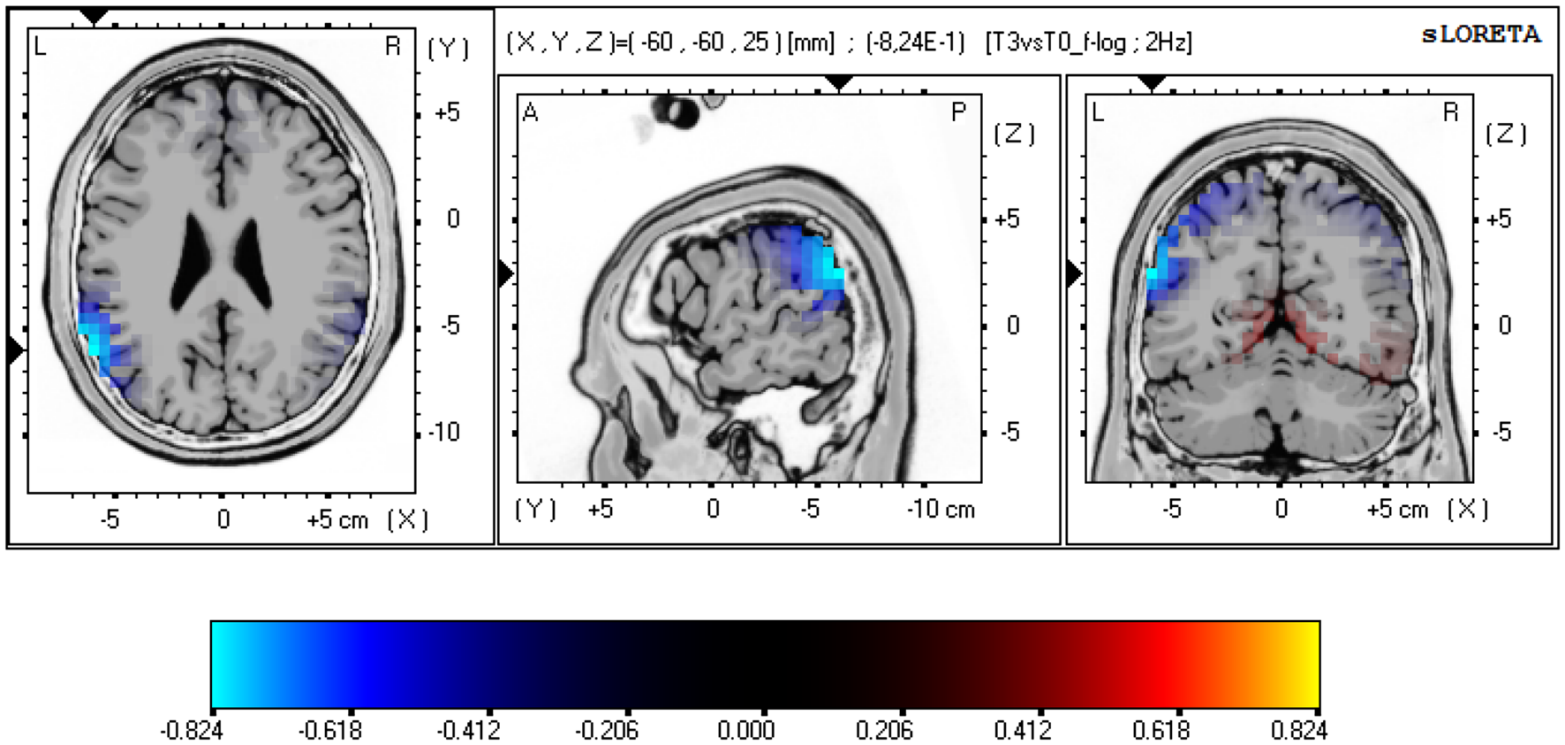
*sLORETA slice viewer, T3 vs. T0: LORETA statistical maps of the alpha-1-frequency band;* statistical non-parametric mapping (SnPM) of within-subject comparisons were performed to compare the current density distributions between T3 (13 to 15 weeks after the marathon) and T0 (T0 = 14 to 4 days prior to the marathon); no significant frequency changes were detected. A statistical trend was detected in form of reduction in alpha-1 power in T3 when compared to T0, Maximum current density differences were localized in the left temporal cortex, (*xyz* = − 60, − 60, 25, BA 39); the color bar represents the log-F-ratio value for each voxel

The following three comparisons were performed: T-1 vs. T0 (representing the effects of training for MA), T2 vs. T0 (corresponding to subacute effects of MA) and T3 vs. T0 (illustrating long-term effects of MA and recovery). Current source density analysis was performed in 3-D Talairach/MNI space (Talairach 1988) using the standardized low-resolution electromagnetic tomography (sLORETA, Pascual-Marqui 2002, The KEY Institute for Brain-Mind Research, Zurich, Switzerland). The first version of LORETA (Pascual-Marqui et al. 1994) has been validated broadly using fMRI (Mulert et al. 2004), PET (Zumsteg et al. 2005) and intracerebral recordings (Zumsteg et al. 2006). The version used in our study (sLORETA, (Pascual-Marqui 2002)) is an advanced version of LORETA and estimates the current source density distribution and source localization in 6.239 cortical gray matter voxels, with a cubic voxel size of 5 mm^3^. For the comparisons across measurements, the sLORETA statistical nonparametric mapping tool (SnPM) was used, based on a paired voxel-by-voxel log-F ratio test using 5.000 randomizations (Villafaina et al. 2019), and included the following frequency bands: delta (1.5–6 Hz), theta (6.5–8.0 Hz), alpha1 (8.5–10 Hz), alpha2 (10.5–12.0 Hz), beta1 (12.5–18.0 Hz), beta2 (18.5–21.0 Hz), beta3 (21.5–30.0 Hz), total power (1.5-30 Hz). Statistical significance levels were set to threshold *p* < 0.05 and threshold *p* < 0.10 was considered as a nonsignificant trend-level finding. The null hypothesis of no activation anywhere in the brain was rejected if at least one test value was above the critical threshold for *p* = 0.05 (Eugene et al. 2015; Horacek et al. 2007). 3D, functional sLORETA images (Figs. 2–4) were calculated representing the electrical current density changes of each voxel in the neuroanatomic Talairach/MNI space (Keeser et al. 2011; Villafaina et al. 2019), corresponding to the estimated neuronal generators of brain activity within each frequency band (Frei et al. 2001). This methodology corrects for multiple testing (i.e., for the collection of tests performed for all electrodes and voxels, and for all time samples. Owing to the nonparametric nature of the method, its validity need not rely on any assumption of Gaussianity (Methods: Statistical analysis of sLORETA / eLORETA 2023; Pascual-Marqui 2002).

## Results

### Demographics

Of the 30 enrolled subjects that participated in the T-1 visit (26 males, mean age = 44.6 years, SD = 9.5), 7 subjects (5 males) dropped out at various points during the study, due to time constraints, as well as medical issues (internal or orthopedic diseases), resulting in 26 subjects (23 males) whose EEG data were included in at least one of the three EEG comparisons and 23 subjects (21 males) completing all four visits of the study. Descriptive and anthropometric data of all subjects who were included in at least one EEG comparison (*N* = 26) are shown in Table 1.

### EEG results

#### Effects of marathon training (T-1 vs. T0)

sLORETA nonparametric mapping (SnPM) was applied to determine and localize changes in frequency bands. The T-1 vs. T0 comparison (*N* = 26) showed no significant differences in delta (log-F ratio = − 0.270), theta (log-F ratio = 0.313), alpha1 (log-F ratio = 0.402), alpha2 (log-F ratio = − 0.447), beta1 (log-F ratio = 0.240), beta2 (log-F ratio = 0.214), beta3 (log-F ratio = 0.318), or total power (log-F ratio = 0. 0.202), (threshold log-F ratio for *p* < 0.05 =  ± 0.682).

#### Post-marathon vs. pre-marathon (T2 vs. T0)

sLORETA nonparametric mapping (SnPM) was applied to determine and localize changes in frequency bands. The T2 vs. T0 comparison showed reduced alpha-2 (10.5–12.0 Hz) power as well as reduced delta (1.5–6 Hz) power in the post-MA condition (T2) as compared to the pre-MA condition (T0). The maximal difference in alpha-2 power was detected in the right frontal gyrus (N = 24, Brodmann area (BA) 8, MNI coordinates: *x* = 5, *y* = 35, *z* = 55, log-F ratio = − 0.705, threshold log-F ratio =  ± 0.685, *p* < 0.05) (Fig. 2, Table 2). The maximal difference in delta power was detected bilaterally in the frontal gyrus (N = 24, BA 6, MNI coordinates: *x* = 40, *y* = 0, *z* = 50, log-F ratio = − 0.699, threshold log-F ratio =  ± 0.685, *p* < 0.05) (Fig. 3, Table 2). The other frequency bands (theta, alpha1, beta1, beta2, beta3, total power) showed no significant differences.

**Table 2:** Statistical non-parametric comparisons between current source density values of the different visits, using sLORETA

	Region	XYZ (MNI)			Brodmann area	Log-F ratio values
T2 vs. T0 (post/pre MA)						
Alpha2 (10.5–12.0 Hz)	Frontal gyrus	5	35	55	8	−0.705*
Delta (1.5–6 Hz)	Frontal gyrus	40	0	50	6	−0.699*
T3 vs. T0 (recovery/pre MA)						
Alpha1 (8.5–10 Hz)	Temporal lobe	−60	−60	25	39	−0.824^†^

*p value < 0.05

^†^*p* value < 0.10

#### Long-term follow-up (T3 vs. T0)

The T3 vs. T0 comparison showed no significant differences in electrical cortical activity (delta: log-F ratio = − 0.711, theta: log-F ratio = − 0.747, alpha1: log-F ratio = − 0.824, alpha2: log-F ratio = − 0.761, beta1: log-F ratio = − 0.700, beta2: log-F ratio = − 0.589, beta3: log-F ratio = − 0.597, total power: log-F ratio = − 0.637, threshold log-F ratio for *p* < 0.05 =  ± 0.828). A statistical trend of reduced alpha-1 (8.5–10 Hz) power was detected in the recovery condition (T3) as compared to the pre-MA condition (T0). The maximum current density differences were detected in the left temporal cortex, BA 39, MNI coordinates: *x* = − 60, *y* = − 60, *z* = 25, *N* = 23, log-F ratio = − 0.824, threshold log-F ratio =  ± 0.805, *p* < 0.10) (Fig. 4, Table 2).

## Discussion

In this study we investigated the effects of moderate intensity PA (preparation for a marathon), strenuous and prolonged PA (MA) and subacute and long-term follow-up on the electrocortical activity of healthy adults in four different phases during training for and recovery from MA. We detected a significant reduction in alpha-2 (10.5–12.0 Hz) power as well as a reduction in delta (1.5–6 Hz) power in the post-MA condition (T2) when compared to the pre-MA condition (T0). When we compared the recovery condition (T3) with baseline (T0), no significant differences in EEG frequency bands were found.

J. Crabbe and R. Dishman reported in their quantitative synthesis a frequently observed increased activity, or frontal hemispheric asymmetry, in the alpha frequency band during and shortly after moderate PA (Crabbe and Dishman 2004). The included studies had significant differences in study design (duration, intensity of PA, concrete timing of the EEG). To avoid reduced comparability resulting from unidentical recording settings, EEG recordings were not performed immediately after MA at the marathon site; therefore, we could not confirm or refute this finding. Our post-MA recordings took place 1–6 days after MA (T2), a phase that reflects subacute effects of MA training and completion.

### Alpha frequency

There are several possible underlying mechanisms for a subacutely reduced alpha frequency band after PA, some of which can be interpreted as counterregulatory effects following a possibly initially increased alpha frequency, as described by other investigators (Stock et al. 1996; Crabbe and Dishman 2004; Krause et al. 1983; Gutmann et al. 2015; Lindgren et al. 1999; Nielsen et al. 2001; Youngstedt et al. 1993 FEB 01:). Discussed underlying components for a PA-induced increase in alpha frequency included thermoregulatory mechanisms, which in turn play a complex mediating role in mood changes (Crabbe and Dishman 2004; Youngstedt et al. 1993 FEB 01:), neurochemical changes (Crabbe and Dishman 2004; Fumoto et al. 2010), increased somatosensory afferents (Crabbe and Dishman 2004; Krause et al. 1983; Youngstedt et al. 1993 FEB 01:) and a high level of arousal and preparedness for external input (Gutmann et al. 2015).

Some previous studies described this acute frequency increase as indicative of central fatigue (Krause et al. 1983), relaxation (Boutcher et al. 1988) or anxiolysis (Petruzzello and Landers 1994). Some authors even postulated that the preexisting fitness level might determine whether an increase in alpha activity indicated exhaustion or relaxation (Boutcher et al. 1988). These effects can be linked to findings associating increased alpha activity with a high level of somatosensory afferents (SSA) induced by strenuous muscle work (Youngstedt et al. 1993 FEB 01:), thereby suggesting that increased SSA can both lead to exhaustion and relaxation, both traceable through elevated alpha activity. When the intensity of SSA has drastically decreased (T2) after maintaining a high level for a significant period (MA), it is likely that counterregulatory mechanisms counteracting the aforementioned effects take place, resulting in a reduction in the alpha frequency band, decreased central fatigue and subsided anxiolysis. The acute anxiolytic effects of PA have also been linked to hypothalamic regulatory circuits postulating similar underlying mechanisms between thermal application and body movement (thermogenic hypothesis) (Youngstedt et al. 1993 FEB 01:). However, the results in this area have been greatly heterogeneous, which may be a consequence of methodological inconsistencies in the studies (DeBoer et al. 2012). Normalization of body temperature after increased body temperature during MA and associated hypothalamic circuits could play an important role in these subacute effects.

PA-induced improvements in mood and mental wellbeing have numerously been described (Dunn and Jewell 2010; Hartescu et al. 2015; Pelletier et al. 2017; Roeh A, 2020; Wipfli et al. 2008), including reduction in depressive symptoms and increase in positive affects in marathon runners when compared to sedentary controls (Roeh et al. 2020). Neurochemical changes are a major component underlying exercise-induced electrocortical adaptations. Correlations between increased alpha power, mood changes, activation and increased oxygenation in the prefrontal cortex (PFC) and elevated serotonin concentration (5-hydroxytryptamine, 5-HT) after PA have previously been described (Fumoto et al. 2010). Our observed post-MA subacute reduction in alpha-2 power was also detected in the PFC (BA 8), an area that plays a major role in cognitive functions and regulation of emotion (Davidson 2001). Our study did not include measurements of serotonin levels, but PA induces increases in serotonin levels as previously described (Fumoto et al. 2010; Medica et al. 2020). Thus, if elevated alpha power is linked to increased serotonin levels after PA cessation, a secondary reduction in alpha frequency a few days after MA could be associated with a compensatory downregulation of serotonin production. Neurochemical confounders are, however, not limited to serotonin, as links have also been described between high (nor-)epinephrine levels and elevated alpha power (Stock et al. 1996). In addition to localization, the hemispheric asymmetry (right > left) in the effect also indicates a connection to mood alterations. There is evidence linking frontal asymmetry, especially in the alpha frequency band, with the effects of neural circuits of affectivity (e.g., Petruzzello and Landers 1994, Davidson 2001, Coan and Allen 2003). There is also evidence linking mood improvements after PA with changes in functional connectivity between the dorsolateral prefrontal cortex (dlPFC) and the temporal region (TMP), two important cortical structures involved in shaping mood, further supporting the affective component in these mechanisms (Ligeza et al. 2021). Our reported effect may be a correlate to complex changes in functional connectivity, which might be verified in further studies.

### Delta frequency

In addition to the reduction in the alpha-frequency band, a reduction in the delta frequency band could be detected post-MA (T2) when compared with T0. Changes in delta activity can often be correlated with neurological pathologies, e.g., various forms of intermittent rhythmic delta activity (IRDA) in epilepsy (temporal IRDA; TIRDA) or encephalopathy (frontal IRDA; FIRDA). There are very limited and inconsistent results on changes in delta activity as an effect of PA (i.e. increase directly after moderate PA in Mechau et al. (Mechau et al. 1998) and Stock et al. (Stock et al. 1996), decrease in Oda et al. (Shiro Oda et al. 1999).

A growing body of evidence proves the effects of PA on cognitive functions such as processing speed, reaction time, memory, attention, planning, and behavioral inhibition (Chang et al. 2012, 2014; Gao et al. 2021; Lambourne and Tomporowski 2010; Tomporowski 2003; Tsai et al. 2014; Weng et al. 2015). One recent study showed improved reaction time in a vigilance task session when a 15-min cycling exercise was inserted compared to the no-intervention session, linking these findings to differences in information processing between the groups and complex changes in functional connectivity induced by PA (Gao et al. 2021). Another recent study detected improvements in EEG slowness (delta waves) as a result of both one session and after six weeks of aerobic exercise while also demonstrating improvements in cognitive functions (using mini-mental state examination (MMSE), Montreal cognitive assessment (MoCA), and trail making test (TMT A and B)), suggesting a link between changes in delta activity and changes in cognition (Amjad et al. 2019). These findings substantiate a link between improvements in cognition, changes in functional connectivity and alterations in delta frequency after PA. Another study reported selected reductions in theta power after PA while also reporting improvements in cognitive functions, proving the heterogeneity in these effects (Devilbiss et al. 2019).

Reductions in the delta frequency band have also been reported as effects of brain stimulation methods, such as transcranial direct current stimulation (tDCS) over the PFC (Keeser et al. 2011; Ulrich Palm et al. 2009). Since tDCS is a plasticity-inducing method and changes in frequency bands could display correlates of this plasticity, the hypothesis arises that running a marathon can induce similar effects and that reduction in delta activity induced by PA and tDCS could be explained by common mechanisms. As tDCS has a positive influence on cognition (Polania et al. 2011) but also may relieve depressive symptoms in depression (Bennabi and Haffen 2018; Fregni et al. 2021; Keeser et al. 2011), this observation contributes to the understanding of EEG measurements in combination with neuroplasticity. These findings could be applied to modifications of cognition as well as in the treatment of depression. Future studies should conduct more precise comparisons between tDCS- and PA-induced EEG effects, which will enable a more precise statement about the similarities of the two interventions.

One limitation of our study is the missing EEG recordings at T1 (directly post-MA). We wanted to measure a naturalistic design with a regular marathon run outside a laboratory to obtain real-world data with better transferability. EEG recordings at the marathon site would not achieve the quality standards of the laboratory recordings (light and sound attenuation, similar recording times between subjects) and would therefore be accompanied by too many confounding variables. Another limitation is the difference in the dates of the EEG recordings at each visit (i.e., 4–14 days prior to MA (T0), 1–6 days after MA (T2) and 13–15 weeks after MA (T3)), with higher variability. This also depicts a naturalistic design but reduces comparability. We aimed at measuring daytime consistent throughout the study period; therefore, we could not measure all participants in one or two days. Our study included only four women, which to some extent originates in the underrepresentation of women in marathon runs. Future studies should try to include more women in similar studies and the effect of gender as a covariate should also be taken into account in the statistical model. Finally, to further substantiate a link between changes in alpha frequency and neurochemical confounders (serotonin, (nor-)epinephrine), future studies in this field should include measurements of blood levels of relevant neurotransmitters and simultaneous EEG recordings around marathon running.

In summary, this study showed for the first time that strenuous and prolonged PA (MA) leads to subacute changes in electrocortical activity, presumably representing alterations in neuroplasticity. Specifically, reduced alpha-2 and delta powers were measured a few days after a MA. Possible underlying mechanisms include subcortical (e.g., hypothalamic) regulatory circuits, hormonal and neurochemical changes, complex modulation of affectivity, and changes in functional connectivity. Moreover, these effects were most profound in frontal cortical regions, which play a major role in affect, cognition and decision making. At long-term follow-up, these effects diminished, suggesting that no long-term persistent neuroplasticity effects can be detected by a marathon run using EEG.

## Data Availability

The data that support the findings of this study are available upon request from the corresponding author.

## Declarations

### Trial registration

http://apps.who.int/trialsearch/Trial2.aspx?TrialID=DRKS00012496.
